# Special Relativity Derived from Spacetime Magma

**DOI:** 10.1371/journal.pone.0100583

**Published:** 2014-06-24

**Authors:** Fred Greensite

**Affiliations:** Department of Radiological Sciences, University of California Irvine, Irvine, California, United States of America; University of Nottingham, United Kingdom

## Abstract

We present a derivation of relativistic spacetime largely untethered from specific physical considerations, in constrast to the many physically-based derivations that have appeared in the last few decades. The argument proceeds from the inherent magma (groupoid) existing on the union of spacetime frame components 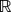 and Euclidean 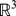 which is consistent with an “inversion symmetry” constraint from which the Minkowski norm results. In this context, the latter is also characterized as one member of a class of “inverse norms” which play major roles with respect to various unital 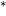-algebras more generally.

## Introduction

In an effort to acquire a deeper understanding of its necessity, in recent years there have been a number of alternative derivations of relativistic spacetime using differing combinations of physical principles distinct from those employed in the original electrodynamical presentation. These have variously included arguments related to the consistency of Newton's First Law and spacetime isotropy with non-instantaneous interaction propagation [1], a probability distribution resulting from quantum fluctuations of the spacetime metric [2], presence or absence of practicable prediction based on the relevant resulting well-posed versus ill-posed field equations [3], requirements for the existence of observed elementary particles and dynamics in the context of basic quantum mechanical considerations [4], implications of electric/magnetic reciprocity [5], and the effective ill-posedness of the Einstein field equations unless the Minkowski metric pertains locally [6] (with the above citations representing only a sample of the relevant work published on this topic). We here augment these efforts by presenting a derivation unencumbered by such physical principles, relying instead on more primitive mathematical considerations - in particular, a symmetry deriving from the inherent magma (or groupoid) implied by a spacetime frame. Perhaps this can be seen as support for recent sentiments that reality accessible to physical investigation is little more than mathematical structure [7], [8]. It also allows alternative placement of relativistic spacetime as prior to, rather than deriving from, the various physical formalisms. A further consequence of our presentation (developed in some detail) is the attendant understanding of the Minkowski norm as one member of a class of “inverse norms”, which are important features of various unital 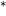-algebras more generally.

## Results

### A magma-derived spacetime algebra

A magma is simply a set with a product that maps any pair of elements of the set into the set [9].

#### Derivation of the Minkowski metric from the magma of space and time

It would seem pointless to pursue physics in the absence of an assumption confirming the possibility of a local “observer” within a space, capable of making a series of “physical predictions” and executing a series of subsequent measurements evaluating those predictions. Notwithstanding speculations concerning a universe with multiple time dimensions, and (indeed) timeless formulations [10], we will interpret the prior sentence as indicating an observer-dependent co-dimension-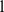 foliation of spacetime with the leaves parameterized by “time” (this is formalized as an axiom in [5]). More specifically, we assume that “phenomena” in affine spacetime can be described in the context of any embedded coordinate frame that indexes the points as 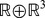 where 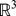 is Euclidean. This is in line with the latter's role as the global coordinate system underlying all established (i.e., experimentally supported) nongravitational physics, and as the local coordinate system in general relativity (i.e., 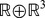 is the coordinate system of the tangent space at each point of the classical spacetime pseudo-Riemannian manifold). Since 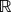 and 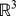 are 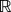-vector spaces, and 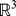 is endowed with the Euclidean inner product, it follows that 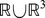 is a magma, i.e., the magma product 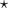 is given by the three preexisting vector space product operations, so that for 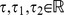, and 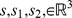 we have 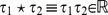, 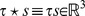, and 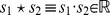. Thus, from their associations with 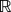 and Euclidean 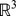, time and space are already thoroughly linked (or jumbled together) prior to taking their direct sum. Via the obvious vector space isomorphisms, the spacetime points comprising the union of the two spacetime frame components 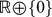, 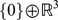, is then also a magma, with 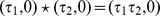, 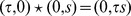, and 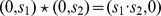. The magma exists for any spacetime frame of the specified form, and for any pair of spacetime points there are such frames with respect to which their magma product can be calculated. Elevating the magma products to the status of phenomena (because the quotient, as the operation inverse to the product, is intrinsic to description of the physical phenomenon of velocity), the product of any two spacetime points should be able to be calculated in some sense in the context of any frame (in accordance with the assumption made earlier in this paragraph; see also the subsection following the next one). This would be equivalent to associating each spacetime point with a map of spacetime to itself. It is significant that a linear map is defined by the product of any fixed point of the magma with the points of either of the magma's linear subsets (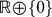 or 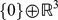). There is a unique elaboration of the product operation such that this linearity property pertains over all spacetime points in the context of the frame - i.e., so that the magma product generalizes in a manner consistent with the linear properties of the space 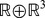, and so that spacetime nonlinearities do not exist (e.g., consistent with the prior notion that the spacetime frame be ‘flat’). From this linearity requirement, the product of any two points with respect to a frame of the above type conforms to the distributive law, and is given by 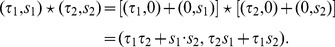
(1)


In this way, the point set subject to a spacetime frame is an algebra (a slightly different perspective on this is given in the Discussion section). Note that this is distinct from other algebras that have been associated with relativistic spacetime [11]-[13], which are Clifford algebras and do not address the question of deriving the Minkowski norm from comparable first principles (this is another issue considered a bit further in the Discussion section). The magma-derived algebra we have identified above is commutative and nonassociative (as opposed to Clifford Algebras, which are associative and usually noncommutative), and is a Spin Factor Jordan algebra.

The *sum* operation of a vector space allows the definition of linear transformations, and the Euclidean norm implies linear isometries as given by the orthogonal group. In this case, equality of magnitude of two points of 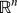 is invariant under the application of any member of 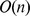. The symmetry group 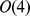 is not compelling for 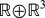, since time and space axes do not necessarily seem interchangeable based on their respective envisioned dynamical roles (and the asymmetry suggested by the co-dimension 1 foliation of spacetime). However, the additional *product* operation of the spacetime magma-derived algebra allows recognition of an alternative constraint - the invariance of equality of magnitude of two points under (multiplicative) inversion rather than (orthogonal group) rotation-reflection. This constraint becomes quite attractive in light of inherent magma-derived algebra inversion symmetry, as noted below.

Explicitly, we make the following assumptions concerning the magnitude function 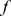 on the entirety of the spacetime magma-derived algebra:

1. *Positivity on the units*. 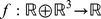 is such that 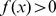 if 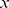 is a unit of the algebra (i.e., if 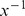 exists),
2. *Homogeneity*. For 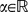, 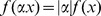,

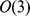
 *-invariance*. 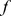 is invariant under an orthogonal transformation of 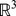, i.e., 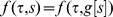, for 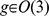 with 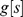 being the point of 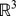 that 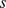 is mapped to under the action of 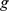,
4. *Magnitude of the identity element*. 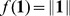, where 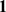 is the identity element of the magma-derived algebra (so that 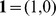 in this case), and 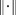 is the Euclidean norm (so that 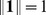),
5. *Equality of magnitude of units implies equality of magnitude of their inverses*. For any units 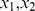 in the magma-derived algebra, if 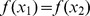 then 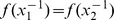.

The above assumptions imply that 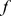 is the Minkowski norm. That is, Positivity and Homogeneity imply that for any unit 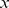 in the algebra there exists 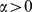 such that 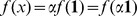. “Invariance of equality of magnitude under inversion” (assumption 5 above) then implies that for any unit of the magma-derived algebra, 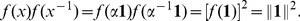
(2)


Thus, 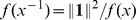, and so inversion of a point is accompanied by inversion of its magnitude. Since the equation 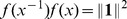 is invariant under the transformation 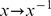, we consider that equation to express “inversion symmetry”. On our magma-derived algebra the inverse of a unit is evidently given by 
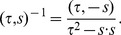
(3)


Using (2), the Homogeneity property, and the Euclidean 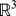 reflection symmetry (i.e., 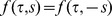, from assumption 3 above), we then have 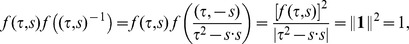

*and so* 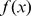 *is the Minkowski norm* (and the latter might be more properly labeled a magmitude rather than a magnitude since there are nonzero elements for which the Minkowski norm is zero).

The square of the Minkowski norm is a quadratic form on domains where 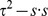 has a consistent sign, and the Minkowski inner product follows in the usual way that inner products are derived from quadratic forms. That is, we have the Polarization Identity: if 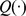 is a quadratic form on a linear space, and 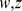 members of this space, then we define 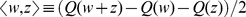, where 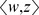 is then seen to be a symmetric bilinear form. Taking 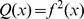, we obtain the (pseudo-)inner product 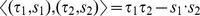, which defines the Minkowski metric. Alternatively, the Minkowski inner product arises spontaneously from the quotient of two points, as is clear from (4) below (and more perspective on this is given in the first subsection of the Discussion section) - so it is not strictly necessary to apply the Polarization Identity.

Thus, one may conclude that Minkowski spacetime is a consequence of an inversion symmetry stemming from a magma implied by the inherent products linking 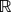 and Euclidean 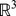, ultimately deriving from identification of an observer's time and space with the latter vector spaces.

#### Mathematical implications of the magma-derived algebra

As alluded to just prior to the above enumeration of the assumptions of the derivation of the Minkowski norm, there are inherent features of our spacetime magma-derived algebra that relate to inversion symmetry, and serve to further justify assumption 5 of the above derivation of the Minkowski norm:

First, 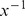 is an irrotational vector field. Equivalently, considered as a 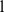-form (covector field), 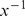 is curl-free (or more precisely, the 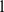-form 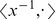 is curl-free, where 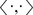 is now the Euclidean inner product), and thus is the exterior derivative of a 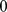-form, the latter scalar function being the integral of 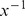, which on a simply connected domain comprising only units and containing 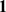 is the path independent 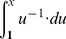 up to a constant (where, unless specified otherwise, “

” is the Euclidean inner product, in this case four-dimensional).

Second, we have the equation 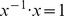, so a unit and its multiplicative inverse have a kind of reciprocity with respect to the Euclidean inner product. The appearance of this equation is unchanged by the transformation 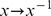, so we consider it to express “inversion symmetry”. Since that equation indicates that the inner product of a point with its inverse is constant over spacetime, two points cannot be distinguished based on their (Euclidean, i.e, with fully symmetric terms) inner products with their respective inverses. Since a magnitude function is a one-dimensional representation of features of the space (and should not add “extraneous information”), it would be expected that a magnitude function faithful to the algebra should also embody the above lack of distinction between units. That is, it should have the feature that points are not distinguishable from the product of their magnitude with the magnitude of their inverse, 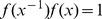 (equation (2)), from which the Minkowski norm follows.

Now, because it is commutative, the above spacetime magma-derived algebra is trivially a 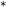-algebra where the involution 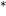 is the identity (in the Discussion section, an alternative involution on this algebra is considered). This is important to recognize so that we can place the Minkowski norm in the context of analogous structures that occur more generally. So, to begin, we recall that a 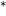-algebra is simply an algebra that is associated with an (anti-)involution. That is, in addition to the algebra 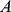, there is an operation 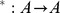 such that for 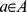 we have 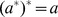, and for 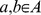 we have 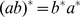. Familiar examples of 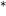-algebras include any commutative algebra (trivially, with 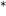 being the identity), the complex numbers (where the involution could be either the identity or the complex conjugate operation), the quaternions (where 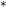 is a generalization of the complex conjugate), and the algebras of real or complex matrices (with 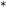 being the transpose or Hermitian transpose, respectively). Clifford algebras also become 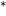-algebras once a particular (anti-)involution is selected.

In the context of the magma-derived algebra's role as a 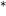-algebra, the two inversion features noted in the two paragraphs before last then translate into assertions that 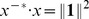, and 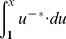 is path independent on a simply connected domain of units containing 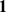 and is thus the integral of curl-free 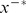. We will show that if the latter features hold for a particular real 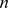-dimensional unital 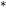-algebra then we can always obtain a positive-homogeneous magnitude function (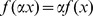 for 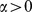) with level sets given by the level sets of the integral of (co)vector field 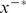. We refer to these functions as “inverse norms” in contrast to the Euclidean norm, a positive-homogeneous magnitude function with level sets given by the level sets of the integral of (co)vector field 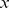. While the Euclidean norm solves the equation 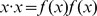 with 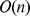-symmetric left-hand-side, we shall see that an inverse norm solves the 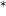-inverse-symmetric equation 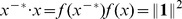. Examples of inverse norms include the Euclidean norm on the Cayley-Dickson algebras (the reals, the complex numbers, the quaternions, the octonions, the sedenions, 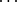), the Minkowski norm on the Spin Factor Jordan algebras, and the 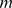-th root of the determinant multiplied by 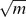 on the algebra of real 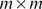 matrices. From this standpoint, the latter three structures are the same thing expressed in the context of different algebras.

Inverse norms share with Euclidean norms the feature that almost all points can be decomposed as the product of a magnitude function with a gradient of the magnitude function, where the gradient of the magnitude function itself has unit magnitude (if 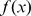 is taken to be the Euclidean norm, then 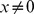 is decomposed as 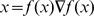 with 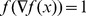; for inverse norms, see Theorem 3 below).

Before placing the Minkowski norm in the more general context of inverse norms, we will consider the physical meaning of the magma-derived spacetime algebra.

#### Physical implications of the magma-derived algebra

As was our intent, we have derived the Minkowski norm independent of usual physical arguments - i.e., we have used only the entities of time and space, which can also be thought of in purely mathematical terms (as justified by physical experiments). Once the Minkowski norm is derived, it has (of course) well known profound physical implications such as the relativity of simultaneity, the contraction of length in the direction of velocity, and (once energy and momentum are introduced) the equivalence of mass and energy. However, although we have forwarded a derivation that is ‘prior to’ subsequently introduced physics (of the type cited in the Introduction), the mathematical structure underlying this derivation has intrinsic physical meaning.

Velocity is one of the most basic of physical concepts, and it is expressed as a quotient of quantities related strictly to space (in the numerator) and strictly to time (in the denominator). From a general perspective, this structure in isolation could be considered a little unsatisfactory (or incomplete): If from an observer's viewpoint we divide a space quantity 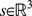 by a time quantity 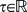 to obtain a velocity 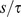, we know from aforementioned isomorphisms that this can be equivalently thought of as point 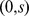 on the frame's space component divided by point 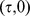 on the frame's time component. The magma product (pre-existing on the union of the frame components) then provides the agreeable 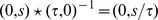 as an analogue of the previously computed velocity 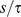. But how is it that only points residing on frame components are able to be divided? A general perspective recognizing the validity of all inertial frames “demands” that we be able to divide any spacetime unit by any other spacetime unit within a specific spacetime frame (otherwise it must be conceded that frame component points are very special, while a default viewpoint would suggest they are not so special). Indeed, the physical concept of velocity as a quotient of points on respective frame components motivates us to not only supply the implied division process for general spacetime points (that are units), but also to do this in a manner that is Lorentz covariant. In fact, this is precisely what the inherent spacetime magma-derived algebra accomplishes. Recalling (1) and (3), if we divide 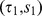 by 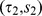, we obtain 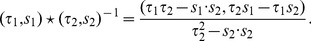
(4)


On the right-hand-side above, the denominator and the two components of the numerator are evidently Lorentz covariant expressions. Thus, the act of computation of the quotient of two points is Lorentz covariant (however, the *result* of the computation is a single point which, of course, is not Lorentz covariant - e.g., velocity is relative to a frame).

Furthermore, the operation of division in the magma-derived spacetime algebra has a definite physical meaning apart from its Lorentz covariant treatment of the ratios from which a velocity is computed. The first component of the ordered pair on the right-hand-side of (4) is the Minkowski inner product of the two points - reflecting the deviation of the two points from being pseudo-orthogonal (points that are pseudo-orthogonal lie on the respective components of a possible spacetime frame). In contrast, the second component of the ordered pair reflects the deviation of the two points from being linearly dependent. If the two points are linearly dependent, then there is an inertial frame such that they and the origin of the spacetime frame are events that occur at the same point in space in the context of that inertial frame. Note that this second component is antisymmetric and bilinear. Analogous to outer products in Clifford algebras, this latter expression can be taken to be the *Minkowski outer product*. This perspective is further developed in the Discussion section. At this point, we summarize these physical aspects as follows:

- The magma-derived algebra satisfies the need to provide a Lorentz covariant expression for the operation of division of spacetime units - an implied generalization of the procedure of computing a velocity.
- The output of the magma-derived algebra's division operation is a point with complementary relativistic components - the first being proportional to the Minkowski inner product, and the second being proportional to the Minkowski outer product - these having manifestly evident physical interpretations.

Finally, we also note that the Lorentz covariant expression for velocity given by the right-hand-side of (4) itself provides an alternative route for derivation of the Minkowski metric from spacetime magma, and that route will be explored elsewhere.

### The Minkowski norm in context: Inversion symmetry and inverse norms on unital 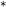-algebras

It is now our objective to formalize the understanding of the Minkowski norm as an example of a structure that occurs as a fundamental feature in a variety of mathematical contexts that are both familiar and have seemingly little in common with Special Relativity. The implication will be that it is not at all surprising that the Minkowski norm arises in spacetime.

We have previously noted that

- The Euclidean norm on 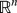 is a positive-homogeneous magnitude function 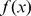 with level sets given by the level sets of the integral of (co)vector field 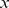, and which solves the equation 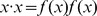 with 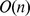-symmetric left-hand-side.
- In the context of our magma-derived spacetime 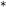-algebra, the Minkowski norm is a positive-homogeneous magnitude function 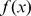 with level sets given by the level sets of the integral of (co)vector field 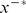, and which solves the 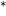-inverse-symmetric equation 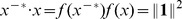.
- Two features of the magma-derived algebra are that on an appropriate domain of units we have 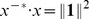, and 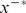 is irrotational. The latter express the “inversion symmetry” of the algebra, and are precisely the features needed for the existence of the important analogues of the Minkowski norm on other 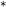-algebras.

Our use of the term “inversion symmetry” refers to a discrete symmetry. That is, the relevant group action leaves an *equation* 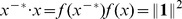 unchanged for each 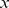 in a domain of units. The group in question has two elements: the identity (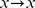, for each 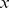 in the domain), and the operation of 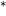-inversion (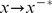, for each 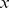 in the domain). In contrast, it is the *solution* to the equation over the specified domain of units (the inverse norm) that is expected to be invariant under the action of a Lie group.

To construct a magnitude function on a unital 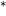-algebra mimicking the Euclidean norm but based on vector field 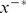 rather than vector field 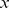, it is first required that 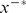 be a gradient, since the level sets of the positive-homogeneous magnitude function are to be determined by the level sets of the integral of that (co)vector field. To embody the property of “invariance of equality of magnitude under 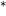-inversion”, it is necessary for 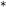-inversion to map magnitude function level sets to magnitude function level sets. Equivalently, given that the magnitude function is positive-homogeneous, the requirement is that the magnitude function level set containing 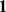 be invariant under 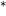-inversion of the space. The latter also imply that the magnitude function values of an element and its 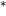-inverse are reciprocal.

Formalizing these statements, we have

#### Definition 1

*A real finite dimensional unital 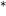-algebra has inversion symmetry if on some neighborhood of 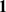 the vector field 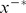 is a gradient and*

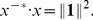
(5)


The inversion symmetry is further characterized by

#### Theorem 1

*A real unital 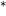-algebra has inversion symmetry if and only if there is a simply connected neighborhood 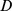 of 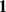 where a function 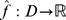 with 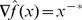 is such that the set of level sets of 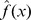 and the set of level sets of 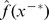 are the same set.*

#### Definition 2

*For a function 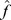 and neighborhood 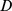 of 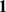 relevant to an algebra with inversion symmetry as in Theorem 1, an inverse norm is a positive-homogeneous function 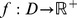 having the same level sets as 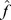 and such that*

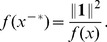
(6)


#### Theorem 2

*An algebra with inversion symmetry has a unique inverse norm.*

The above theorems are corollaries of the following, which provides another characterization of an inverse norm.

#### Theorem 3

*A real finite dimensional unital 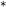-algebra has inversion symmetry if and only if on this algebra there exists a simply connected domain 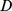 containing 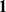 with 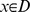 implying both 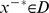 and 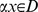 for any real 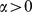, such that there exists 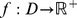, with*

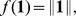

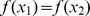
 *implies* 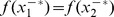,
- *any element of 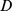 can be decomposed as*

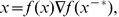
(7)
*with*

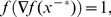
(8)
*where* 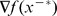 *indicates that the gradient* 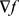 *is evaluated at the point* 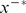.

#### Corollary 1

*The function 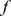 specified by Theorem 3 is the inverse norm associated with the given algebra.*

In summary: a 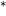-algebra on which 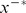 is an irrotational vector field, has inversion symmetry if the expression 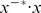 is invariant, i.e., constant. This constant must necessarily be 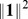 considering the case of 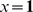. Since the expression 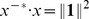 doesn't change under the transformation 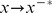, we refer to that property as expressing inversion symmetry. An inverse norm 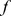 on the algebra is required to be positive homogenous (like norms in general), but be developed from the integral of 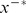 (in contrast to the Euclidean norm, which is positive homogeneous but develops from the integral of 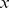), and it must solve the 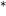-inverse symmetric functional equation, 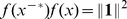. For the magma-derived algebra of the last section, 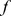 is then the Minkowski norm. But the inverse norm turns out to be an important structure in the setting of other algebras having nothing to do with Special Relativity. Understanding of Special Relativity is deepened by recognizing its underlying kinship with these different areas, and that is the reason we have introduced the above definitions and theorems. For example, when considered from the standpoint of its inherent algebraic structure as ultimately expressed by the inverse norm, the magma-derived algebra of Special Relativity is kin to any of the Cayley-Dixon algebras, and indeed to the 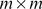 real matrix algebra itself. From that perspective, the appearance of the Minkowski norm in spacetime is not strange at all, given that the physical entity of velocity suggests (as we have argued) that spacetime should be viewed as an algebra.

Thus, inversion symmetry (Definition 1) is the feature of the magma-derived algebra which implies that a spacetime frame has a Minkowski norm - the prototype of an inverse norm. More generally, inversion symmetry is the thing that is necessary and sufficient for an inverse norm to be defined on an algebra, and is thereby responsible for recognition of a revealing structural similarity between Special Relativity and other seemingly remote mathematical settings.

### Proof of the Theorems

As an aid in identifying the key steps in the proof of Theorem 3, we will italicize certain sentences. A general idea of the strategy of the proof can be obtained by initially concentrating on these italicized sentences.

#### Proof of Theorem 3, Necessity

For the Necessity portion of the proof, inversion symmetry is assumed, and we must show that it implies all statement following the “if and only if” clause in the statement of the theorem.

*Inversion symmetry implies existence of a simply connected domain containing 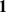 on which 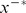 is a gradient and 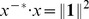. Without loss of the prior two properties, this domain can be enlarged to meet the specifications of 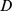 in the theorem statement.* That is, we first enlarge the original domain to consist of all non-origin points lying on the rays from the origin through any point of the original domain. This new neighborhood 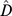 is still simply connected and it is clear that 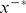 is a gradient and 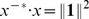 on 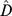. Consider 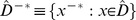. The operation 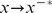 maps open rays to open rays. Since that operation is a diffeomorphism between 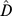 and 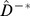, the set 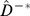 is simply connected, is a union of open rays, and shares at least one of these rays with 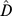 (the ray through 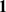). It follows that 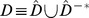 is simply connected, and 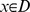 implies both 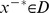 and 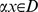 for 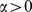.

*Thus, there exists a function 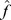 on 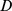 such that*

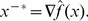
(9)


*Selection of a particular constant of integration fixes 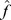. Since 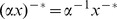, applying (9) twice yields*

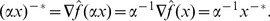
(10)


Now consider 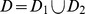 where 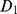 is the subset of 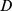 whose members can be written as a positive multiple of some member of the level set of 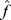 that contains 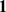, and 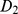 is its compliment in 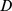. If 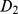 is nonempty, then since 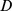 is connected there is an 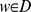 such that every neighborhood of 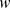 contains a point of 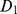 and a point of 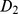. The ray from the origin through 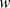 must necessarily be tangent to the level set of 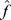 containing 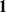 at some point 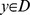 (otherwise, there either won't be rays from the origin passing arbitrarily near 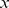 that intersect the level set containing 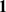 or there won't be rays arbitrarily near 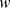 not intersecting the level set containing 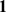). But then the gradient of 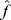 at 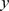 is normal to the ray from the origin passing through 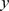. Since 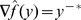, this means that 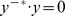, which contradicts (5). Since 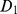 cannot be empty (it contains the open ray from the origin containing 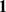), it follows that 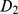 is empty. Thus, any 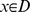 can be written as 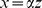 for some 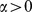 and some 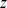 in the level set of 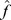 containing 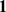. We can also see that this decomposition is unique. That is, suppose for some 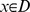 we have 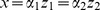 with 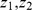 distinct points on the level set of 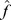 containing 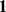. Since the gradient vectors 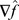 are parallel to each other at each point of the open ray from the origin through 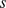 (as is evident from (10)), 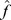 is strictly increasing, strictly decreasing, or constant, along the open ray. And yet, 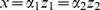 implies that the ray intersects the level set containing 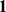 at least twice, which is a contradiction if 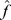 is either strictly increasing or strictly decreasing. Thus, 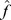 would be required to be constant along the open ray containing 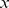, implying that the gradient at 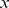 (which is 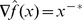) is normal to this ray - and so its inner product with 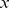 is zero, again contradicting (5). Thus, the decomposition 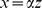 is unique.

For a fixed choice of 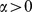, 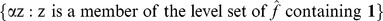
(11)is a level set of 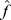, because the second equality in (10) implies that the gradient of 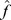 at any point 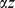 of the set (11) is proportional to the gradient at the corresponding open ray point 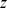 on the level set containing 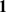. *Thus, the level sets of* 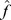 *are each a dilation of the level set containing* 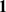.

*We now construct a positive-homogeneous function* 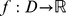 *which will have the same level sets as* 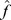. First, we specify that all points 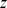 in the level set of 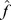 containing 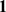 are given the value 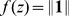. As we have seen, any point 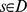 can be uniquely written as 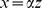 with 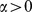 and 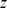 in the level set of 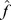 containing 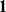. We thus complete the definition of 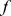 on points of 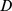 via 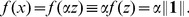
(12)


The functions 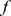 and 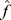 are seen to have the same level sets in 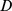, since their level sets are dilations of the level set of 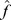 containing 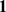.

*Since by assumption 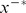 is a gradient on 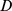, the level set of 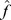 (and 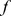) containing 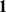 is smooth in 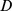, and by construction 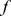 is differentiable on 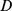. For 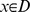, we claim that for* 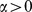, 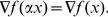
(13)


This follows because the level sets of 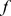 are dilations of each other via (12), and so the set of directional derivatives of 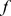 at a point 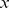 will be proportional to the set of directional derivatives of 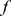 at a point 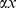. But (12) implies that for any point on the open ray defined by the origin and any of the points 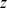 on the level set containing 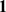, the directional derivative of 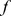 with respect to a unit vector along the open ray is constant everywhere on the open ray (a nonzero constant, because of (5) and (9)) - thus, the other directional derivatives, and the gradient itself, must also be constant along the points of the ray, and (13) follows.

Now consider 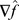 at points of 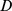 on the open ray defined by 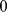 and 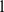. From (9), we have that the gradient of 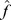 at points of this ray is directed along the ray. Since 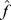 and 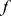 have the same level sets, it follows that 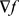 at points on this same ray also is directed along this ray. The norm of 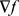 at points on this ray is thus the value of the directional derivative specified by the vector of norm 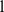 in the direction of the ray. The unit norm vector in this direction is 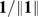. But 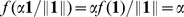, which follows from (12) and our earlier specification of the value of points on the level set containing 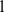, where in particular, 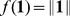. Hence, the derivative of 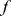 in the direction given by unit vector 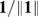 is 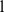, and we then have 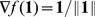. Equation (9) implies that 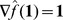. Thus, 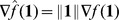. Since the level sets of 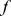 are also level sets of 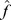, the gradients of 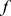 and 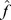 must be proportional at each point of a level set. *Hence, for* 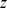 *on the level set containing* 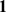, *the last equation implies *

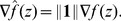
(14)


*So, for 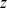 on the level set containing 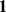, (12), (13), (14), and (9), imply that*

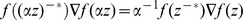


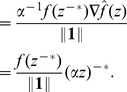
(15)


*We will now show that 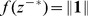 for any 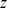 in the level set containing 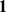, from which (15) will imply (7). *First, from (9), for 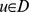, 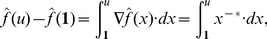
(16)since 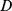 is simply connected. Similarly, since by assumption 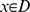 implies 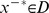, 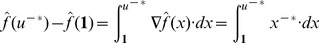
(17)

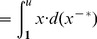
(18)

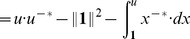
(19)

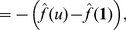
(20)where (18) follows from the change of variable 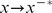, (19) follows from integration by parts, and (20) follows from (16) and (5).

Equation (20) implies that 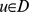 is on the level set of 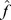 containing 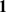 if and only if 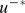 is on the level set of 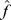 containing 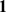. Since in 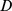 the level set of 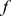 containing 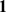 is the same as the level set of 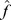 containing 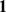, we then have that 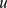 is on the level set of 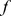 containing 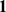 if and only if 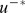 is on the level set of 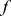 containing 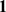. This means that the term multiplying 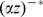 on the right-hand-side of (15) is unity (since we now have 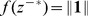 because 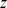 is on the level set containing 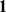 which is now seen to imply that 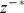 is on the level set containing 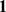 and we have previously defined 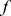 to have the value 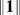 on the level set containing 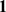). As we have seen earlier, any 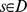 can be written uniquely as 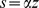 for some 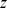 on the level set of 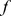 containing 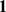 and some 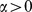. *Consequently, (15) implies that for* 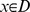, 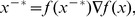
(21)
*which is equivalent to (7). Applying 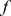 to both sides of (7) and then using the second equality in (12) we obtain (8)*.

*Finally, (7) and (21) imply*

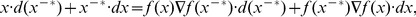
(22)
*which is equivalent to*

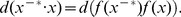
(23)


*Since by assumption* 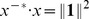, *the left-hand-side above is zero, and so* 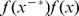 *is constant on* 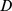. *This means that for* 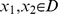 *we have* 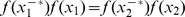. *Thus* 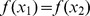 *implies* 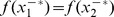. *Q.E.D.*

#### Proof of Theorem 3, Sufficiency

For the Sufficiency portion of the proof, all of the statements following the theorem's “if and only if” clause are assumed, and inversion symmetry must be derived from them - i.e., it must be shown that 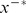 is a gradient on 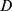 and 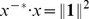.

Equation (7) implies (21) and together these imply (22) and (23). Integrating both sides of (23) on any path in simply connected 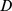 from 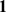 to 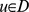, and noting that by assumption 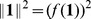, *we obtain *

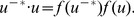
(24)


*Since* 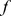 *is strictly positive on* 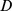 *and* 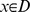 *implies* 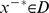, *this means that* 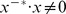.

*Now we establish positive-homogeneity, i.e., for* 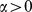, 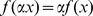. Applying (7) twice, we have 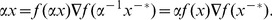
(25)


Equation (8) then gives 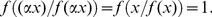
(26)


If 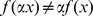 then (26) implies that there are two different points on the ray from zero through 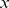 having the same magnitude. But according to (25), 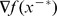 and 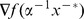 are parallel, and so 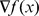 and 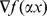 are parallel for any 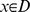. This means that for points of 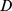 on the ray through 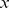 it must be that 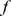 is strictly increasing or strictly decreasing or constant. Since there are two points on the ray having the same value of 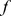, it follows that 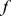 must be constant on the ray, so that 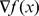 is normal to the ray from 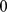 through 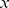, i.e., 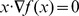. But from (21), 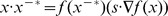 and we have already noted that (24) indicates that the left-hand-side of this equation is nonzero, contradicting the prior sentence. Thus, it is required that 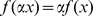.

*Since by assumption 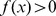 in 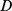, for any particular choice of 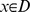 we can find an 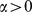 such that 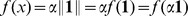, where the last equality follows from positive-homogeneity. Since the value of 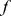 is thus the same at 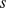 and 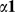, by assumption we have 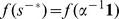. Once again using positive-homogeneity, we obtain the functional equation*

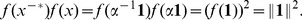
(27)


*Equation (5*) then follows from (24).

*We now only need show that* 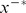 *is a gradient on* 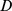. Equations (7) and (8) indicate that every 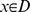 can be written as 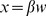 with 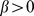 and 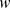 in the level set of 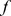 with the value 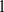. This decomposition must be unique because otherwise we would have 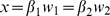 with 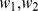 on the level set of 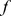 with value 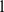. But after applying 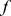 to both sides of the second equality in the last sentence, positive-homogeneity would imply 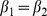, which would then imply 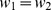. By positive-homogeneity, the level set of 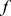 containing 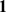 is just a dilation of the level set having value 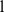. Thus, we can uniquely write 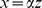 with 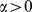 and 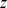 on the level set of 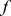 containing 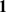 (the points of this set have value 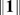 by assumption). Hence, we can define a new function 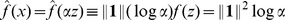. Note that 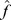 has the same level sets as 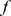, so the gradients of 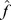 and 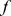 are proportional at the points of any level set. Directional derivatives along the ray 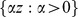 result from 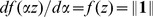 and 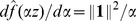 (using positive-homogeneity for the first equation and our above specification of 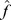 for the last equation). Thus, 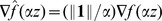. Since (27) implies that 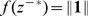, we have that (21) (equivalent to (7)), positive-homogeneity, and the equation of the last sentence, imply 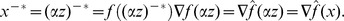



The above indicates that 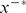 is a gradient. *Q.E.D.*

#### Proof of Theorem 1

As regards *Sufficiency*, by assumption there is a function 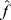 on a simply connected neighborhood 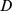 of 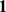 whose gradient is 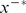, i.e., 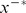 is a gradient on 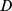. Since it is also assumed that 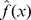 and 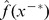 have the same set of level sets, 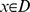 implies 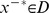. Equations (16) through (19) then follow on this domain. Let 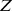 be the set of points on the level set of 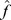 containing 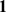. For 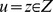, (16) implies that the integral on the right-hand-side of (19) is zero. Since 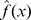 and 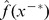 have the same set of level sets, the level set of 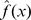 containing 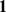 (the set 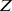) is the same as the level set of 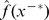 containing 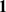. This implies that for 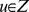 the left-hand-side of (17) is zero. Thus, for 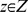, (19) implies 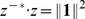. Since 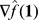 is normal to 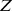 at 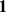, there is a neighborhood 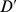 of 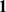 such that every point of this neighborhood is on a ray from the origin through some point 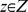, and so each 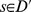 can be written as 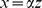 with 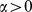, 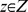. We then have for any 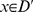, 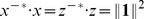. Thus, 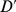 is the neighborhood of 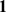 required by Definition 1 for inversion symmetry to be present.

As regards *Necessity*, inversion symmetry implies all of the statements of Theorem 3 following the “if and only if” clause. This implies equations (16) through (20), which imply that the set of level sets of 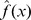 and the set of level sets of 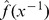 are the same on 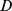. *Q.E.D.*

#### Proof of Theorem 2

Inversion symmetry implies the statements of Theorem 3 following the “if and only if” clause (since the latter theorem has been proved), including the requisite simply connected domain 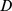 of that theorem. Therefore, we may use the arguments from the Proof of Sufficiency portion of the proof of Theorem 3, where it was shown that 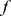 is a positive-homogeneous function satisfying (27), i.e., (6). The final paragraph of that Proof of Sufficiency demonstrates the required relationship of 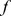 to a function 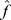 with 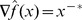. Thus, 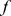 fulfills the requirements of Definition 2.

The level sets of the integral of 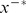 are unique regardless of the constant of integration. These are also the level sets of the inverse norm, whose level set containing 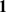 is assigned the value 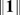. The values of the remainder of the level sets of the inverse norm are then uniquely determined by the positive-homogeneity condition. Thus, the inverse norm is unique. *Q.E.D.*

The above proof of Theorem 2 also establishes Corollary 1.

## Discussion

### Modifications of the magma-derived spacetime 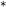-algebra

While the product of two spacetime points (equation (1)) gives no immediate hint of what magnitude function should be associated with the magma-derived algebra, the inverse operation (division) does, as is clear from our remarks following the appearance of equation (4). Furthermore, the numerator on the right-hand-side of (4) by itself is a bilinear product on 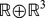, 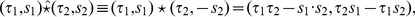
(28)defining an algebra with a product given by the differences (rather than the sums as in (1)) of all magma-generating products across components of the two spacetime points. This new algebra is nonunital, noncommutative, nonassociative, and is not a 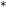-algebra. Essentially, it is a magma on the entirety of 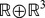 that is consistent with the latter's linear structure. But this new magma has some nice features. The first component of the product on the right-hand-side of (28) is a symmetric bilinear form, and so we can define an inner product as 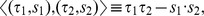
which is, of course, the Minkowski inner product. The second component of the product on the right-hand-side of (28) is antisymmetric and bilinear, and so we can define an outer product as 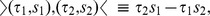
which we call the *Minkowski outer product*. Thus, 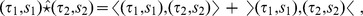
and 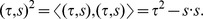



The latter implies a magnitude function (or magmitude function) as given by the Minkowski norm. Thus, this magma-algebra (i.e., a nonunital non-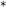-algebra with a nonassociative and noncommutative product) has a Lorentz invariant product, implies a “Minkowski outer product”, and implies a Minkowski metric. We have already noted that the first component of the product is the deviation of two points from being pseudo-orthogonal, and the second component expresses the deviation of two points from being linearly dependent - a juxtaposition of complementary geometric features of the two points considered in tandem.

We can also observe that the involution selected for the original magma-derived algebra (with product (1)) need not be the identity. Thus, we also have a 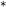-algebra if the involution is defined by 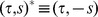. It is easy to verify that the Minkowski norm still satisfies (5) through (8) given the understanding that the inner product and gradient are consistent with this choice of 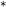. That is, satisfaction of (5) now requires that “

” be the Minkowski inner product, i.e., 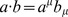 where the contravariant vector is determined by a metric tensor implied by the involution. Similarly, (7) and (8) require that “
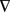
” be the four-gradient 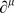. This suggests a liberalization of Definition 1 wherein the choice of inner product is made such that (5) holds. Nevertheless, we are also free to suggest that the involution be chosen such that the latter equation holds with the maximally symmetric Euclidean inner product. In either case, using the above alternative involution we are at least able to write 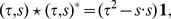
while retaining the structure of a commutative unital 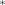-algebra.

The above alternative product and involution choices notwithstanding, it should be clear that the original magma-derived spacetime algebra can be understood as *inherent* in the vector space 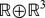 when 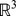 is Euclidean and 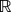, 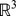 are distinguished (as opposed to 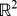, 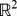) by their respective identification with an observer's “time” and “space” - the single underlying physical assumption. An analogy with vector space 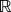 is apt. A vector space is associated with a field such that there is a product of the members of the field with the vector space members that maps into the vector space. The field is typically looked at (necessarily) as an entity distinct from the elements of the vector space. But if the vector space is 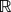, then the nominally extraneous field is isomorphic to the vector space and need not be considered extraneous since the product of a field element and a vector space element can in this case be also thought of as a product between vector space elements, meaning that this particular 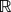-vector space is inherently an algebra. The essential feature in recognizing this triviality is the isomorphism between the field and the vector space. Though slightly more complicated, the statement that ‘the union of the frame components of 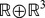 (where 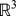 is Euclidean) is inherently a magma’ is analogous, through the respective frame component isomorphisms with 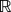 and Euclidean 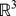. Applying the linearity condition (spacetime should be ‘flat’), the frame itself is then recognized as an algebra.

### The exceptional status of spacetime: are inverse-normed algebras rare?

As we have already observed, considered from the standpoint of its inherent algebraic structure as ultimately expressed by the inverse norm, the magma-derived algebra inherent in Special Relativity has a fundamental similarity to any of the Cayley-Dixon algebras, and indeed to the 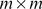 real matrix algebra itself. From this standpoint, Lorentzian geometry is not so strange.

However, based on the criteria specified in Definition 1, it might also be suggested that inverse normed algebras are uncommon. It is true that the Cayley-Dickson algebras all have an inverse norm (given by the Euclidean norm). But although an inverse norm exists on the algebra of real 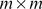 matrices (as 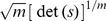), its subalgebras (i.e., the real finite-dimensional associative algebras) do not in general have inverse norms. For example, for the two dimensional real matrix algebra with the usual matrix product and elements of the form 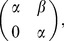
(29)there is no involution that can allow satisfaction of either criterion of Definition 1. While the Spin Factor Jordan algebras have inverse norms (as the Minkowski norm), none of the other formally real Jordan algebras do. Although the algebra built on the real 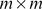 matrices where the product is given by the anticommutator multiplied by one-half has an inverse norm (as 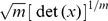), the Lie algebra built on the real 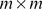 matrices (where the product is given by the commutator) does not have a unital hull [14] associated with an inverse norm. This incidentally motivates the question of enumerating all inverse normed algebras. Another task would be the identification of all algebras not satisfying the criteria of Definition 1 but still admitting a solution to the 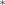-inverse symmetric functional equation 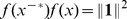.

### A comparison of spacetime algebras as regards derivation of the Minkowski norm

A very successful spacetime algebra (STA) is described in [11] (as a “unified mathematical language for physics”), this being the Clifford algebra 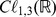. However, this algebra is chosen because its associated quadratic form is consistent with the Lorentz signature. Thus, it does not have a role in derivation of the Minkowski norm.

The elegant formulation of [13] forwards the planar-spacetime algebra 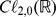, and is demonstrated to be successful in description of various physical processes. A consequence of this choice is the identification of time with a spatial bivector, rather than introducing an extraneous time dimension (such as Minkowski's 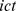). However, selection of this Clifford algebra is made precisely because the square of the sum of a scalar and a bivector is consistent with a Lorentz signature. Thus, this approach cannot be considered to address the derivation of the Minkowski norm, although it certainly pertains to its interpretation.

The Algebra of Physical Space (APS) [12] is another successful spacetime algebra, this time utilizing 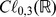. It has been suggested that it is particularly natural, since it is the Clifford algebra arising from Euclidean 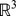. However, this selection is not unique. That algebra's multivectors, multiplication rules, and further imposed Clifford conjugate, are not already present or uniquely implied by 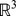 itself (and other algebras could as easily be associated with spacetime). Ultimately, it appears that the motivation for introduction of this algebra relates to the fundamental nature of the Pauli matrices, i.e., this choice of algebra is implied by the formalism of quantum mechanics, and in that regard (insofar as it relates to a “derivation” of the Minkowski norm) this approach has a kinship with references cited in the Introduction. On the other hand, it is interesting to note that although APS is not uniquely implied, 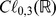 with involution chosen to be the Clifford conjugate satisfies the criteria of Definition 1, and thus has an inverse norm. It is then quickly verified that this inverse norm applied to a paravector yields the Minkowski norm of the paravector.

In contrast to the above Clifford algebras, the magma-derived algebra we forward unambiguously follows from the preexisting linkages between an observer's time and space through their association with 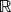 and 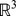. A magma exists as soon as one postulates spacetime as 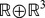 with Euclidean 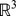 - it is simply an acknowledgement of the products pre-existing on and between the constituent vector spaces. It is an inherent constituent of the frame, whether or not one chooses to examine the implications of that constituent. In our approach (and that of others [5]), time arises from the co-dimension 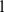 foliation of spacetime that is an underlying axiom corresponding to the series of observations/predictions that characterize human experience.

### Is Lorentzian spacetime geometry prior to the laws of physics, or does it derive from the laws of physics?

The unexpectedness of the Minkowski norm and its consequences have prompted explanations of its necessity, given that the well grounded physical formalisms in place have a reality potentially independent of the prior choice of spacetime signature [1], [2], [4]-[6]. Nevertheless, the particular physical theories employed for this purpose (classical mechanics, quantum field theory, classical electrodynamics, gravitation) arise from highly specific physical observations (so using them leads to arguments that the observed universe implies the Minkowski norm, rather than an independently derived Minkowski norm implies the observed universe). The argument in [3] is more general, suggesting (among other things) that the Lorentzian signature is required so that the equations underlying successful prediction will be well-posed. However, the latter is not a constructive argument, being more along the lines of *reductio ad absurdum* and evidently based on the anthropic principle. It doesn't tell us where the metric comes from, but only that it must be. In contrast, we have shown that spacetime Lorentzian geometry arises naturally/inevitably from basic ingredients of a frame (since time and space are found to be *physically* joined, why wouldn't that be governed by their inherent *mathematical* linkage?), using a symmetry argument that (compared to the anthropic argument) seems more consistent with the manner in which explanations of physical reality are usually developed and interpreted.

Ultimately, our objective has been to derive Lorentzian spacetime geometry from non-physical principles (other than the axiomatic existence of “time” and “space” with respect to an observer and the concept of “velocity”), so that the equations observed in nature in part *follow* from an independent derivation of the Minkowski norm - rather than the other way around as in the work cited above, not to mention that of Einstein and Poincare. This at least serves to place the spacetime metric on a level co-equal with the cited physical principles.
